# Unusual Central Venous Catheter Position in Hemodialysis: Anatomical Considerations

**DOI:** 10.1016/j.xkme.2025.101006

**Published:** 2025-04-16

**Authors:** Hiroki Ito, Takuo Hirose, Takefumi Mori

**Affiliations:** 1 Division of Nephrology and Hypertension, Tohoku Medical and Pharmaceutical University, Sendai, Japan; 2 Division of Integrative Renal Replacement Therapy, Tohoku Medical and Pharmaceutical University, Sendai, Japan

A 90-year-old man with a long history of hypertension presented with creatinine 10.1 mg/dL and pulmonary congestion with pleural effusion on diagnostic imaging. He was diagnosed with end-stage kidney disease due to hypertension, causing volume overload heart failure.

Hemodialysis was initiated via a right internal jugular venous catheter. On catheter occlusion, placement of a new catheter in the left internal jugular vein was performed. The guidewire and catheter insertion proceeded smoothly without resistance, and hemodynamic stability was maintained throughout the procedure. The catheter demonstrated excellent blood flow parameters.

**Figure 1:**
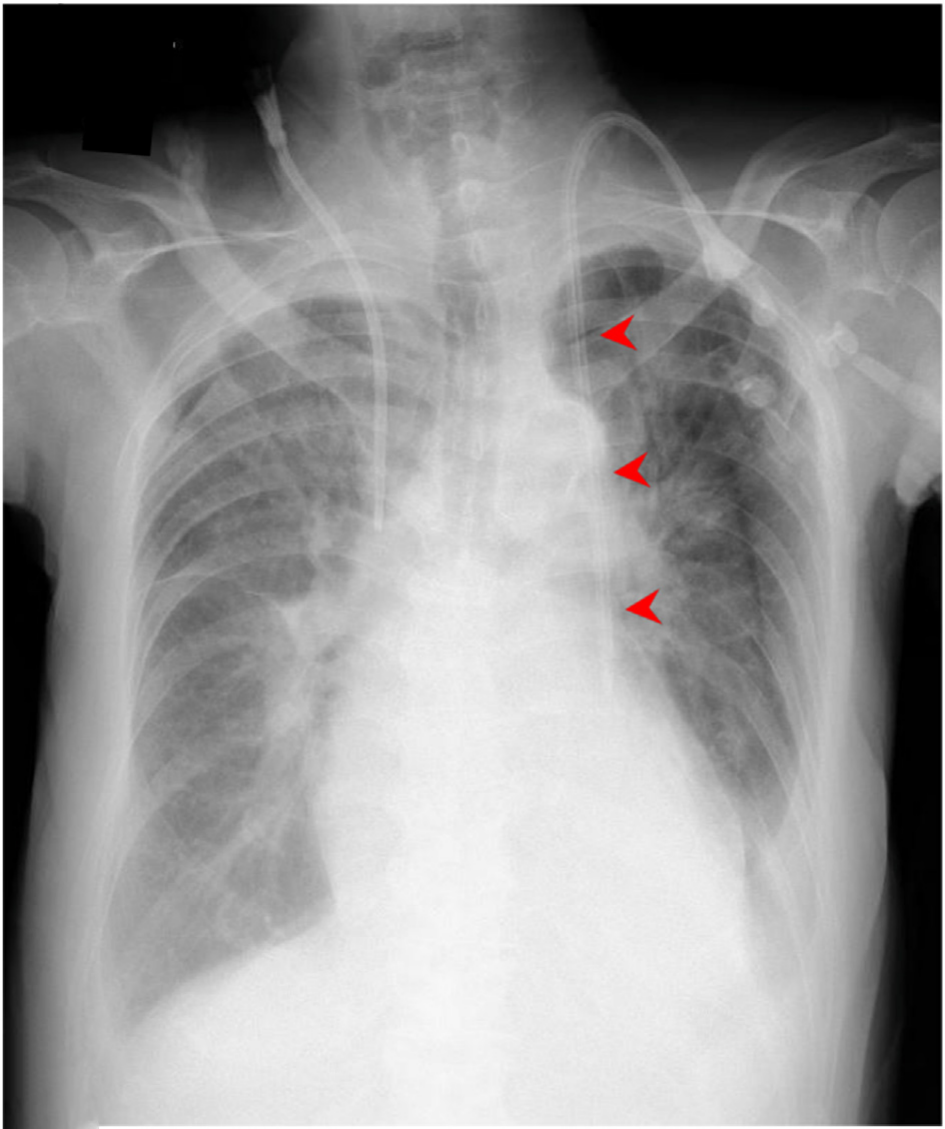
Chest radiograph showing hemodialysis catheter positioned along the left side of the mediastinum (arrow head).

**Figure 2:**
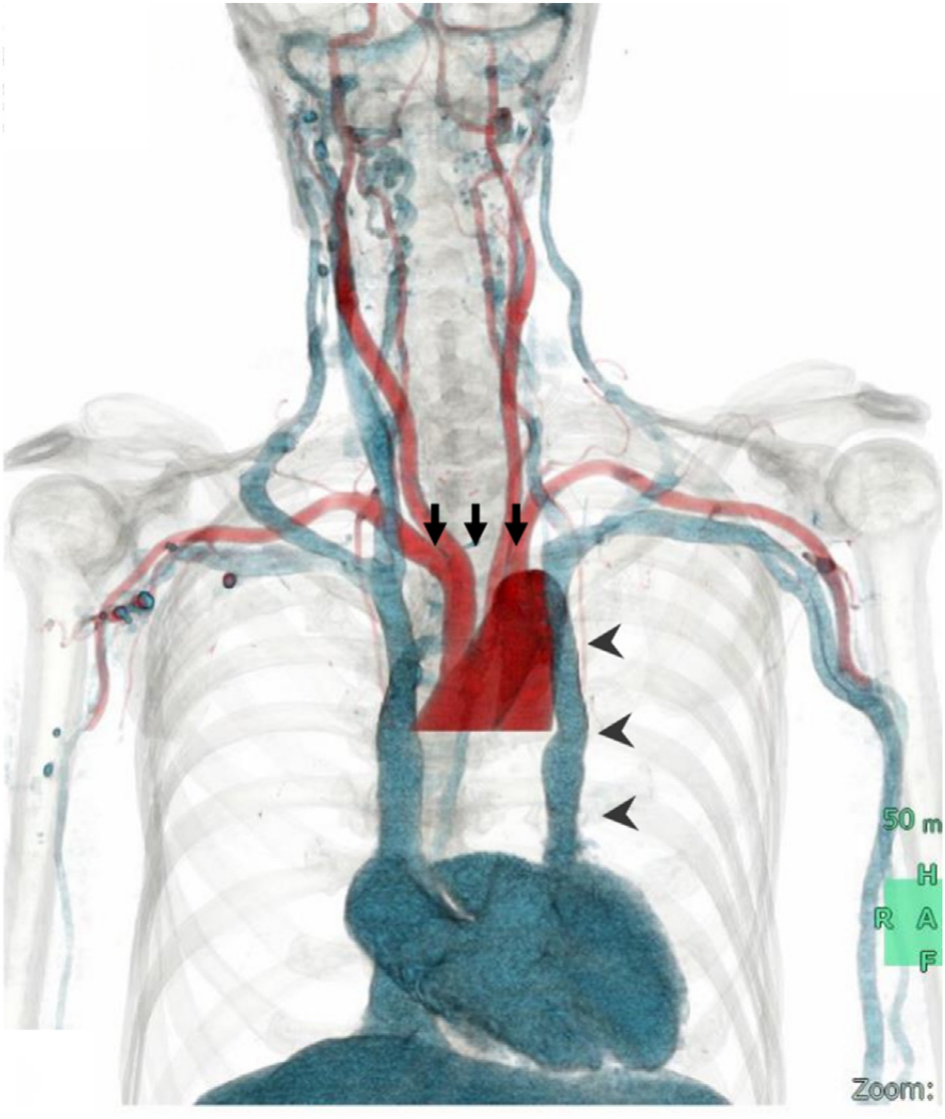
Contrast-enhanced computed tomography demonstrating absence of connection between the right and left brachiocephalic veins (arrow) and presence of left superior vena cava coursing down the left mediastinum and draining into the heart (arrow head).

Postprocedural chest radiography revealed an unusual catheter course along the left mediastinal border (Fig 1). Contrast-enhanced computed tomography demonstrated the absence of the left brachiocephalic vein, with the left internal jugular vein joining the left subclavian vein and descending directly to the heart, confirming persistent left superior vena cava (PLSVC) (Fig 2).

The PLSVC occurs in 0.3%-0.5% of the general population,^1^^,^^2^ with higher prevalence (3%-10%) among patients with congenital heart disease.^3^ This vascular variant typically represents a benign anatomical variation rather than a pathological complication. However, this catheter malposition raises concerns about venous perforation, arterial misplacement, or other congenital cardiovascular abnormalities, which are differential diagnoses. PLSVC is often associated with cardiac/splenic malformations and potential arrhythmias. When PLSVC is suspected, the diagnosis can be confirmed through contrast-enhanced computed tomography, angiography, or transthoracic or transesophageal echocardiography. Recognition of PLSVC is crucial for nephrologists and interventionalists performing central venous access procedures to avoid unnecessary manipulation of correctly positioned catheters within this variant anatomy.
